# Coparenting Closeness and Psychological Distress as Predictors of Life Satisfaction Among Romanian Divorced and Separated Parents

**DOI:** 10.3390/bs16071213

**Published:** 2026-07-17

**Authors:** Maria-Manuela Apostol, Magdalena Iorga, Lidia Liliana Melnic, Camelia Soponaru

**Affiliations:** 1 Department of Behavioral Sciences, Faculty of Medicine, Grigore T. Popa University of Medicine and Pharmacy, 700115 Iasi, Romania; maria_manuela.apostol@umfiasi.ro; 2 Faculty of Psychology and Educational Sciences, Alexandru Ioan Cuza University of Iași, 700554 Iasi, Romania; camelia.soponaru@uaic.ro; 3 Faculty of Psychology, Behavioral and Legal Sciences, Andrei Șaguna University of Constanța, 900196 Constanța, Romania

**Keywords:** coparenting, psychological distress, life satisfaction, divorce, separation, regression analysis

## Abstract

Background: Divorce and separation represent major life transitions involving substantial changes in family organization, interpersonal relationships, and individual functioning. Despite the end of the romantic relationship, parents often remain connected through ongoing coparenting responsibilities, making post-separation adjustment a continuing relational process. The aim of the study was to examine the associations between coparenting dimensions, psychological distress, and life satisfaction among Romanian parents who were divorced, separated, or in the process of separation. Methods: A convenience sample of 206 divorced or separated parents (83.5% women; Mage = 43.39) completed a self-report questionnaire collecting sociodemographic data and identifying scores for coparenting quality of relationship (Coparenting Relationship Scale), psychological distress (Depression Anxiety Stress Scale—DASS-21), and life satisfaction (Satisfaction with Life Scale—SWLS). Results showed that positive coparenting dimensions were positively associated with life satisfaction, whereas conflictual dimensions and psychological distress were negatively associated with well-being indicators. Psychological distress was strongly negatively associated with life satisfaction. In the regression model, only higher coparenting closeness and lower psychological distress emerged as significant predictors of greater life satisfaction. Conclusions: These findings indicate that, when considered simultaneously, the emotional quality of coparenting—particularly closeness—and individual psychological distress are the most relevant predictors of life satisfaction in post-separation parents. The findings suggest that interventions aiming to strengthen coparenting relationships and address psychological distress may be associated with improved well-being among separated or divorced parents. However, these implications should be interpreted considering the cross-sectional design.

## 1. Introduction

Separation and divorce represent major life transitions that involve substantial changes in family organization, interpersonal relationships, and individual functioning (Amato, 2010; Raley & Sweeney, 2020). Although the dissolution of a romantic relationship formally ends the couple subsystem, former partners often remain interconnected through shared parenting responsibilities, resulting in continued interdependence within the post-separation family system (Adamsons & Pasley, 2013; Steinbach, 2019). As a result, post-divorce adjustment extends beyond the termination of the romantic relationship and involves ongoing coordination between parents in relation to childrearing responsibilities.

Coparenting after divorce has increasingly been understood as a central relational mechanism through which separated parents reorganize family functioning and maintain parental responsibilities. Systematic and recent evidence suggests that the quality of post-divorce coparenting is strongly associated with parental adjustment, child well-being, and family functioning (Lamela & Figueiredo, 2016; Teubert & Pinquart, 2010; Akarçay Ulutaş & Taşkıran, 2024; Zhao et al., 2022). In particular, cooperative coparenting and lower interparental conflict appear to be associated with better parental well-being and life satisfaction, whereas conflictual coparenting may contribute to emotional strain and maladaptive post-divorce adjustment (Augustijn, 2023; van Dijk et al., 2020; Ferraro & Lucier-Greer, 2022).

Contemporary research on divorce has increasingly shifted from structural and legal perspectives toward an emphasis on post-separation relational processes (Johnson & Wu, 2002; Kim & McKenry, 2002; Strohschein et al., 2005; Williams & Dunne-Bryant, 2006). From this perspective, post-divorce adjustment is understood as a dynamic process shaped by the quality of interparental interactions, communication patterns, and conflict management between former partners (Amato, 2010; Feinberg, 2003; Lamela et al., 2016). These relational processes have been consistently linked to parental psychological functioning and broader indicators of adjustment following separation (Hald et al., 2023; Lamela et al., 2016).

Family Systems Theory provides a useful conceptual framework for understanding these dynamics (Minuchin, 1985; Cox & Paley, 2003). This perspective posits that families operate as interconnected systems composed of multiple subsystems whose functioning remains interdependent even in the context of structural changes such as divorce (Cox & Paley, 2003; Feinberg, 2003). Accordingly, divorce reorganizes the family system but does not eliminate the functional links between former partners, particularly in relation to shared parenting roles and responsibilities. Within this broader system, the coparenting subsystem represents a central organizational structure in post-separation family life (Feinberg, 2003; McHale & Lindahl, 2011).

Coparenting refers to the ways in which parents coordinate their roles, manage parenting responsibilities, and communicate about child-related decisions outside the romantic relationship context (Feinberg, 2003). This subsystem plays a key role in maintaining continuity in parenting practices and in shaping the emotional and relational environment in which children and parents adapt to family restructuring (Lamela & Figueiredo, 2016; McHale & Lindahl, 2011).

Coparenting is widely conceptualized as a multidimensional construct that includes both cooperative and conflictual processes (Feinberg, 2003; McHale & Lindahl, 2011). These dimensions typically encompass agreement regarding childrearing practices, perceived support between parents, relational closeness, endorsement of the partner’s parenting role, exposure to interparental conflict, and undermining behaviors. Rather than representing a single global evaluation, these dimensions reflect distinct but interrelated aspects of post-separation parenting interactions, which may coexist within the same coparenting relationship (Feinberg, 2003; Rejaän et al., 2022).

The concept of Coparenting Closeness refers to the degree of emotional connection, mutual support, and shared investment between individuals who share parental responsibilities. It does not only refer to the logistical coordination of child rearing, caregiving, and education tasks, but also includes psychological alignment and relational warmth within the coparenting dyad. It is a shared sense of parental efficacy, accompanied by open communication about the child’s well-being, and a perceived solidarity that reinforces the parental authority of each partner. Only by promoting a safe and collaborative environment between parents does coparenting closeness serve as a critical mechanism that directly influences both individual parenting behaviors and overall family functioning (Lamela et al., 2016; Augustijn, 2023; Ferraro & Lucier-Greer, 2022). Recent reviews and meta-analytic evidence further support the multidimensional nature of coparenting and its associations with parental functioning, family adjustment, and child outcomes (Teubert & Pinquart, 2010; Zhao et al., 2022; Akarçay Ulutaş & Taşkıran, 2024).

Empirical evidence indicates that coparenting quality is meaningfully associated with parental psychological adjustment following separation (Lamela et al., 2016; Augustijn, 2023). Positive coparenting processes are generally linked to more adaptive functioning, lower parental stress, and reduced psychological distress, whereas negative coparenting processes are associated with increased emotional strain, poorer adjustment outcomes, and greater vulnerability to psychological difficulties (Lamela et al., 2016; van Dijk et al., 2020; Zhao et al., 2022; Ferraro & Lucier-Greer, 2022). These findings highlight the importance of distinguishing between adaptive and maladaptive dimensions of post-separation parenting relationships.

The Stress Process Model provides a complementary theoretical explanation for these associations (Pearlin et al., 1981; Pearlin, 1999). This framework proposes that chronic interpersonal stressors contribute to psychological distress by increasing emotional burden and reducing coping resources. Within post-divorce contexts, ongoing conflict, lack of coordination, and undermining behaviors may function as persistent stressors that negatively affect psychological functioning. Recent evidence further suggests that coparenting quality is associated not only with parental adjustment and well-being but also with parental burnout and family functioning, highlighting the broader psychological significance of coparenting processes across different developmental stages and family contexts (Zhang & Zhao, 2024).

Divorce and separation are also associated with increased psychological vulnerability, particularly when relational conflict, parenting responsibilities, and economic or social stressors persist after the end of the couple relationship (Sbarra et al., 2015). Previous studies have linked divorce with elevated depressive symptoms, anxiety, stress, and lower psychological well-being (Johnson & Wu, 2002; Kim & McKenry, 2002; Strohschein et al., 2005; Williams & Dunne-Bryant, 2006). Although many individuals gradually adapt to these transitions, persistent interpersonal conflict and parenting-related challenges may prolong psychological difficulties and hinder adjustment (Mandemakers et al., 2010; Hald et al., 2023). Consequently, psychological distress represents a relevant indicator of post-divorce adaptation and an important predictor of broader well-being outcomes.

Psychological distress, commonly conceptualized as a composite of depressive, anxiety, and stress symptoms, represents an indicator of impaired psychological functioning (Lovibond & Lovibond, 1995). Extensive evidence shows that higher levels of psychological distress are associated with lower subjective well-being across populations (Keyes, 2005; Steel et al., 2008). Subjective well-being includes both affective and cognitive components, with life satisfaction representing its cognitive evaluative dimension (Diener et al., 1985, 2018). Consistent with broader family research, parental stress and psychological distress have been repeatedly associated with lower life satisfaction, whereas resilience-related resources may attenuate these negative effects and promote psychological adjustment (Gavín-Chocano et al., 2024; Rusu et al., 2025).

Life satisfaction refers to a global cognitive appraisal of one’s overall quality of life and functioning across domains (Diener et al., 1985, 2018). In post-divorce populations, life satisfaction is shaped by both relational factors, such as coparenting quality, and individual psychological factors, such as psychological distress (Augustijn, 2023; Lamela et al., 2016; Arcangeli & Ejova, 2025).

Longitudinal studies have consistently shown that divorce is associated with significant declines in life satisfaction, particularly around the period of marital dissolution (Lucas, 2005; Doré & Bolger, 2018). Although many individuals experience gradual recovery over time, substantial variability exists in adaptation trajectories, suggesting that relational and psychological resources may play an important role in post-divorce adjustment (Luhmann et al., 2012; van Scheppingen & Leopold, 2020). Similar findings were reported by Preetz (2022), who showed that the dissolution of non-cohabiting relationships was associated with short-term declines in life satisfaction and increased depressive symptoms, although substantial heterogeneity in adaptation trajectories was observed across individuals. More recent evidence indicates that supportive interpersonal relationships, adaptive coping mechanisms, and social support may facilitate recovery and promote higher levels of life satisfaction following separation (Arcangeli & Ejova, 2025; Dierker, 2025).

Consistent literature has shown that, across different countries and cultural contexts, post-divorce life satisfaction is strongly associated with the quality of the coparenting relationship and may function as an important protective factor for both individual and family outcomes. For example, Lamela et al. (2016) showed that Portuguese parents involved in high-conflict coparenting relationships reported significantly lower levels of life satisfaction. In Spain, Pellón et al. (2024) found that anxiety, time elapsed since divorce, frequency of contact with children, and the quality of the relationship with the former partner were associated with psychological and somatic symptomatology. In the United States, intervention programs targeting parents during the early stages of separation have been developed to increase awareness regarding the management of negative psychological outcomes and to promote the best interests of children (Ferraro et al., 2016). Similar findings were reported by Çelik and Nazlı (2025), who demonstrated that support programs for divorced parents significantly improved life satisfaction while reducing symptoms of depression, anxiety, and stress. Taken together, these findings suggest that relational and psychological factors operate simultaneously in shaping post-divorce adaptation, highlighting the importance of examining their unique contributions within multivariable predictive models.

Taken together, these findings suggest that positive coparenting processes have been consistently associated with better parental well-being and may represent an important resilience-related resource following separation and divorce. (Lamela et al., 2016; Augustijn, 2023; Çelik & Nazlı, 2025; Seal et al., 2025).

Despite growing research on post-divorce adjustment, relatively few studies have simultaneously examined the unique contribution of multiple coparenting dimensions and psychological distress to life satisfaction among divorced or separated parents (Lamela et al., 2016; Augustijn, 2023). Most previous studies have focused either on overall indicators of coparenting quality or on individual psychological outcomes rather than examining these domains within an integrated theoretical framework. Moreover, evidence from Eastern European populations remains limited, despite potentially important sociocultural differences in post-divorce family functioning. Guided by Family Systems Theory and the Stress Process Model, the present study addresses this gap by examining the unique associations between multiple dimensions of coparenting, psychological distress, and life satisfaction among Romanian divorced and separated parents.

The present study integrates Family Systems Theory and the Stress Process Model into a unified conceptual framework. Family Systems Theory provides the relational perspective by conceptualizing coparenting as a dynamic subsystem in which the quality of the relationship between former partners is closely linked to broader family functioning and parental adjustment (Minuchin, 1985; Feinberg, 2003). Complementing this perspective, the Stress Process Model explains how persistent relational stressors may be associated with psychological distress and reduced subjective well-being over time (Pearlin et al., 1981; Pearlin, 1999). Together, these frameworks suggest that post-divorce parental adjustment reflects the interaction between relational functioning and individual psychological processes. Together, these complementary frameworks provide the theoretical foundation for the study hypotheses by linking relational family processes with individual psychological functioning and life satisfaction following separation.

Accordingly, the aim of the present study was to examine the unique associations between multiple dimensions of coparenting, psychological distress, and life satisfaction among divorced and separated Romanian parents within this integrated theoretical framework.

The hypotheses of the research are:

**H1.**  *Coparenting dimensions (agreement, support, closeness, endorsement) will be positively associated with life satisfaction.*

**H2.**  *Negative coparenting dimensions (conflict exposure and undermining) and psychological distress will be negatively associated with life satisfaction.*

**H3.**  *Coparenting dimensions and psychological distress will contribute significantly to the prediction of life satisfaction when considered simultaneously.*

**Figure 1:**
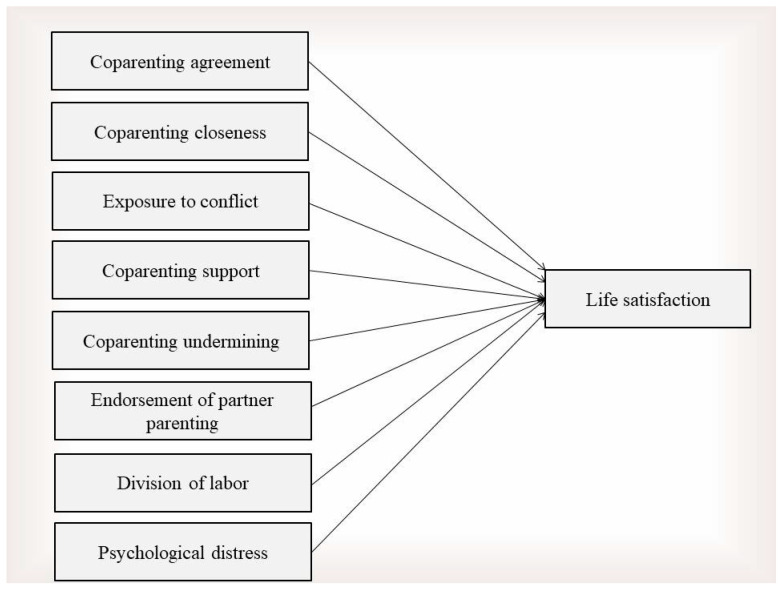
Conceptual framework of the associations between coparenting dimensions, psychological distress, and life satisfaction.

The conceptual framework underlying the present study is presented in Figure 1.

## 2. Materials and Methods

### 2.1. Participants and Procedure

This cross-sectional study was conducted between January and May 2026. Romanian adults were recruited through announcements distributed via social media platforms (Facebook, Instagram, and WhatsApp) and parenting-related online communities targeting divorced or separated parents in Romania. The invitation to participate in the anonymous online survey specified the eligibility criteria, namely being at least 18 years old, being the parent of at least one minor child, and no longer cohabiting with the child’s other parent, regardless of whether the legal separation or divorce proceedings had been finalized. These eligibility criteria were also included as screening questions at the beginning of the questionnaire. The survey was administered using Google Forms. Participation was entirely voluntary, and no financial or material incentives were offered. Before completing the questionnaire, participants received information regarding the purpose of the study, the anonymous and confidential handling of their responses, and their right to decline participation. Electronic informed consent was obtained from all participants before access to the questionnaire was granted. Participants were instructed to answer all items with reference to their current coparenting relationship with the child’s other parent, irrespective of whether they were currently involved in a new romantic relationship.

All questionnaire items were mandatory. Consequently, questionnaires with incomplete responses were not retained for analysis, and no missing-data imputation procedures were required. Prior to the statistical analyses, the dataset was screened for eligibility and completeness. The reported cause of separation was assessed as a single-choice variable, requiring participants to identify the primary perceived reason for the separation. For the regression analyses, gender was dummy coded (0 = female, 1 = male), with female serving as the reference category.

An a priori power analysis using G*Power version 3.1.9.7 (Faul et al., 2009) for a multiple linear regression model with ten predictors indicated that a minimum sample size of approximately 118 participants would be sufficient to detect medium effect sizes (f^2^ = 0.15) with a significance level of α = 0.05 and statistical power of 0.80. The final sample (*N* = 206) exceeded this requirement, ensuring adequate power for the analyses conducted.

### 2.2. Instruments

A questionnaire was constructed for the present study and has two parts. The first one gathered socio-demographic, educational and family-related data. Participants were required to complete a sociodemographic section assessing gender, age, residential area, educational level, marital status (divorced, separated, or in the process of divorce), current romantic relationship status, and child’s living arrangement (i.e., whether the child/children lived with the participant or with the other parent). In addition, participants provided information regarding the causes of their separation or divorce, including infidelity, marital conflict, incompatibility, lack of involvement in family life, alcohol abuse, physical violence, emotional distancing, and other forms of abuse.

The second part contained several psychological instruments:

Coparenting Relationship Scale (CRS; Feinberg et al., 2012) consists of 35 items rated on a 7-point Likert scale ranging from 0 to 6. The Romanian validated version of the Coparenting Relationship Scale was used in the present study (Dumitriu et al., 2022). The first 30 items are rated from 0 (not true of us) to 6 (very true of us), whereas the final five items are rated from 0 (never) to 6 (very often, several times a day). The scale comprises seven dimensions: Coparenting Agreement (e.g., parents agree on issues regarding childrearing), Coparenting Closeness (e.g., parents experience closeness in their parenting relationship), Exposure to Conflict (e.g., the child is exposed to parental conflict), Coparenting Support (e.g., parents support one another in parenting situations), Coparenting Undermining (e.g., one parent undermines the other’s parenting efforts), Endorsement of Partner Parenting (e.g., confidence in the partner’s parenting abilities), and Division of Labor (e.g., satisfaction with the distribution of parenting responsibilities). The CRS operationalizes the multidimensional conceptualization of coparenting proposed by Feinberg (2003) by assessing differentiated domains of coparenting functioning (Feinberg et al., 2012). This multidimensional structure allows the assessment of both positive dimensions of coparenting, including agreement, support, closeness, and endorsement of the partner’s parenting role, and negative dimensions, including exposure to conflict and undermining behaviors. Compared with global measures of parenting quality, the CRS provides a more comprehensive evaluation of the relational processes that characterize post-separation coparenting (Feinberg, 2003; Feinberg et al., 2012; Dumitriu et al., 2022).

Following the assessment of internal consistency, the Division of Labor subscale demonstrated inadequate reliability (Cronbach’s α = 0.18) and was therefore excluded from all subsequent analyses. This decision was based on psychometric considerations, as reliability below commonly accepted standards may compromise the validity and interpretability of statistical findings. Consequently, only the remaining six coparenting dimensions (Agreement, Closeness, Exposure to Conflict, Support, Undermining, and Endorsement of Partner Parenting) were retained for the correlational and regression analyses. The retained subscales demonstrated acceptable to excellent internal consistency: Coparenting Agreement (α = 0.83), Coparenting Closeness (α = 0.75), Exposure to Conflict (α = 0.93), Coparenting Support (α = 0.93), Coparenting Undermining (α = 0.85), and Endorsement of Partner Parenting (α = 0.91).

Psychological distress was assessed using the Depression Anxiety Stress Scales–21 (DASS-21; Lovibond et al., 2011). The instrument consists of 21 items rated on a 4-point Likert scale ranging from 0 (“did not apply to me at all”) to 3 (“applied to me very much or most of the time”). The scale assesses three dimensions of psychological distress: depression, anxiety, and stress. In the present study, the Romanian validated version of the DASS-21 was used. Raw scores were used throughout the analyses. Scores for each subscale were obtained by summing the corresponding item responses, and the overall psychological distress score was calculated by summing the responses to all 21 items. In accordance with the DASS-21 raw scoring procedure, scores were not multiplied by two. Internal consistency was excellent for the overall scale (Cronbach’s α = 0.96, McDonald’s ω = 0.96), while reliability coefficients for the subscales were α = 0.89 and ω = 0.90 for depression, α = 0.909 and ω = 0.90 for anxiety, and α = 0.93 and ω = 0.93 for stress. Higher scores indicate greater levels of psychological distress.

Life satisfaction was assessed using the Satisfaction with Life Scale (SWLS; Diener et al., 1985). The instrument consists of five items rated on a 7-point Likert scale ranging from 1 (“strongly disagree”) to 7 (“strongly agree”). Scores are computed by summing all item responses, with higher scores indicating greater life satisfaction. The SWLS is one of the most widely used measures of the cognitive component of subjective well-being and has demonstrated good psychometric properties across diverse populations (Diener et al., 1985, 2018). In the present study, the scale demonstrated good internal consistency (Cronbach’s α = 0.87).

### 2.3. Data Analysis

Data was analyzed using IBM SPSS Statistics version 27.0. Descriptive statistics were computed to describe the sample characteristics and study variables. Pearson correlation analyses were conducted to examine bivariate associations among coparenting dimensions, psychological distress, and life satisfaction, as well as sociodemographic variables.

To examine the predictive effects of coparenting dimensions and psychological distress on subjective well-being, multiple linear regression analyses were performed. In the main regression model, life satisfaction was entered as the dependent variable, while six coparenting dimensions (agreement, closeness, exposure to conflict, support, undermining, and endorsement of partner parenting), psychological distress, and sociodemographic variables (gender, age, and time since separation/divorce) were entered simultaneously as independent variables.

The control variables included in the regression model (gender, age, and time since separation/divorce) were selected based on previous literature indicating their potential associations with parental adjustment and life satisfaction following separation or divorce. Gender and age have been consistently associated with psychological adjustment in post-divorce research, whereas time since separation/divorce was included because previous studies suggest that psychological adaptation may change over time following marital dissolution. Other potentially relevant variables, such as socioeconomic status, custody arrangements, and the frequency of contact between former partners, were not available in the present dataset and should be considered in future research.

Prior to interpreting the regression results, assumptions of multiple linear regression were evaluated. Multicollinearity was assessed using tolerance values and variance inflation factors (VIF). Additionally, linearity, normality of residuals, homoscedasticity, and independence of errors were examined through standard diagnostic plots and statistical tests.

### 2.4. Ethical Approval

The study protocol was approved by the Ethical Committee of the Faculty of Psychology and Educational Sciences, “Alexandru Ioan Cuza” University of Iași, Romania (Approval No. 28, 18 January 2026). Before starting the survey, the participants were informed about the purpose of the research, the use of the data obtained, and the confidentiality of the data. No incentives were given to the respondents. Those who agreed to participate filled in the online questionnaires.

## 3. Results

### 3.1. Descriptive and Correlation Analysis

A total of 243 individuals completed the survey; however, 37 participants were excluded because they reported not being separated or divorced from the other parent. The final convenience sample comprised 206 parents aged between 28 and 57 years (*M* = 43.39, *SD* = 6.34). The time elapsed since separation or divorce ranged from 1 to 260 months (*M* = 46.93, *SD* = 47.87).

The majority of participants were women (83.5%), and most resided in urban areas (85%). Regarding educational level, the largest proportion held a bachelor’s degree (40.8%), followed by a master’s degree (34%). In terms of marital status, 73.3% were divorced, 12.6% were separated, and 14.1% were in the process of divorce. Most participants reported that the child/children lived with them (83%), while 17% reported that the child/children lived with the other parent. Regarding current romantic status, 58.7% of participants were single, while 41.3% were in a committed romantic relationship.

The most frequently reported causes or claims for separation or divorce included marital conflict, infidelity, and incompatibility. Detailed results are presented in Table 1.

Descriptive statistics for all study variables are presented in Table 2. Skewness and kurtosis values were within acceptable ranges for all variables, suggesting no severe violations of normality assumptions.

Pearson correlation coefficients among study variables are presented in Table 3. As expected, dimensions of positive coparenting (agreement, closeness, support, and endorsement of partner parenting) were significantly and positively intercorrelated, whereas coparenting undermining and exposure to conflict were negatively associated with these adaptive coparenting dimensions.

The analysis of data showed that psychological distress was positively associated with exposure to conflict (*r* = 0.45, *p* < 0.01) and coparenting undermining (*r* = 0.20, *p* < 0.01), while being negatively associated with life satisfaction (*r* = −0.48, *p* < 0.01). Examining the specific dimensions of psychological distress, depression was positively related to exposure to conflict (*r* = 0.40, *p* < 0.01) and coparenting undermining (*r* = 0.18, *p* < 0.05), and negatively associated with coparenting closeness (*r* = −0.15, *p* < 0.05) and life satisfaction (*r* = −0.49, *p* < 0.01). Anxiety showed positive associations with exposure to conflict (*r* = 0.47, *p* < 0.01) and coparenting undermining (*r* = 0.22, *p* < 0.01), while being negatively related to life satisfaction (*r* = −0.40, *p* < 0.01). Similarly, stress was positively associated with exposure to conflict (*r* = 0.41, *p* < 0.01) and coparenting undermining (*r* = 0.16, *p* < 0.05), and negatively associated with life satisfaction (*r* = −0.48, *p* < 0.01). None of these distress dimensions showed significant associations with coparenting agreement, coparenting support, or endorsement of partner parenting.

Life satisfaction was positively related to coparenting agreement (*r* = 0.22, *p* < 0.01), closeness (*r* = 0.44, *p* < 0.01), coparenting support (*r* = 0.34, *p* < 0.01), and endorsement of partner parenting (*r* = 0.33, *p* < 0.01), and negatively related to exposure to conflict (*r* = −0.34, *p* < 0.01) and coparenting undermining (*r* = −0.23, *p* < 0.01).

As expected, the three DASS subscales were strongly intercorrelated and highly correlated with the overall psychological distress score. Psychological distress showed very strong associations with depression (*r* = 0.95, *p* < 0.01), anxiety (*r* = 0.95, *p* < 0.01), and stress (*r* = 0.94, *p* < 0.01), while the subscales themselves were also strongly related (depression–anxiety: *r* = 0.86, *p* < 0.01; depression–stress: *r* = 0.83, *p* < 0.01; anxiety–stress: *r* = 0.84, *p* < 0.01), supporting the coherence of the broader distress construct.

Demographic variables showed generally weak associations with the study variables, with the exception of time since divorce, which was negatively associated with psychological distress (*r* = −0.25, *p* < 0.01), depression (*r* = −0.20, *p* < 0.01), anxiety (*r* = −0.26, *p* < 0.01), and stress (*r* = −0.24, *p* < 0.01). These findings suggest that parents who had been divorced for a longer period of time tended to report lower levels of emotional distress, possibly reflecting gradual psychological adjustment and adaptation to post-divorce circumstances.

### 3.2. Regression Analysis

Prior to conducting the regression analysis, assumptions were checked. Linearity, homoscedasticity, and normality of residuals were assessed through visual inspection of standardized residual plots, normal probability (P–P) plots, and scatterplots of standardized residuals against standardized predicted values, with no substantial deviations observed. Independence of errors was evaluated using the Durbin–Watson statistic (DW = 1.44), indicating no evidence of substantial autocorrelation. Cook’s distance values (maximum = 0.051) and leverage values (maximum = 0.134) did not indicate the presence of influential observations. Multicollinearity diagnostics indicated acceptable tolerance (0.205–0.830) and VIF (1.22–4.88) values. Nevertheless, the positive coparenting dimensions showed moderate to strong intercorrelations, indicating substantial shared variance among these conceptually related constructs. Consequently, the regression coefficients should be interpreted as reflecting the unique contribution of each predictor after accounting for the variance shared with the remaining predictors in the model.

The multiple linear regression analysis was conducted to examine whether six coparenting dimensions (agreement, closeness, exposure to conflict, support, undermining, and endorsement of partner parenting), psychological distress, gender, age, and time since divorce predicted life satisfaction. The overall model was statistically significant, *F*(10, 195) = 13.26, *p* < 0.001, explaining 37.4% of the variance in life satisfaction (*R*^2^ = 0.405, adjusted *R*^2^ = 0.374).

Controlling for all other variables in the model, coparenting closeness emerged as a significant positive predictor of life satisfaction (*β* = 0.43, *p* < 0.001), indicating that higher perceived closeness in the coparenting relationship was associated with higher life satisfaction (see Table 4). Psychological distress also significantly predicted life satisfaction, but in a negative direction (*β* = −0.43, *p* < 0.001), suggesting that higher levels of distress were associated with lower life satisfaction.

None of the remaining predictors reached statistical significance, including coparenting agreement, exposure to conflict, coparenting support, coparenting undermining, endorsement of partner parenting, gender, age, and time since separation/divorce (all *p* > 0.05).

## 4. Discussion

The present study examined the associations between coparenting dimensions, psychological distress, and life satisfaction among parents following or in the process of divorce or separation, as well as the unique contribution of these variables in predicting life satisfaction. Overall, the findings highlight the central role of both relational (coparenting closeness) and individual (psychological distress) factors in explaining life satisfaction.

Demographic variables (age, gender, and time since separation/divorce) did not significantly predict life satisfaction when psychological and coparenting variables were controlled for. One possible explanation is that coparenting closeness functions as an emotional stabilizer within post-divorce family systems, reducing feelings of relational fragmentation and facilitating continuity in parental identity. In this context, relational and psychological processes may play a more central role in shaping life satisfaction than structural or demographic characteristics, consistent with previous findings on post-divorce adaptation (Lamela et al., 2016; Augustijn, 2023).

One possible contextual interpretation is that these findings may partly reflect characteristics of the Romanian sociocultural context, where traditional parental role expectations have been reported in previous research. However, these contextual factors were not directly assessed in the present study and should therefore be interpreted cautiously. Although studies conducted in several European countries have generally reported limited or inconsistent gender differences in post-divorce life satisfaction and adjustment, findings from more traditional and collectivistic cultural contexts suggest that sociocultural factors may substantially influence post-divorce experiences. For example, Badri et al. (2025) reported that divorced women in the United Arab Emirates experienced elevated levels of emotional distress, social disconnection, and reduced mental well-being, partly due to cultural norms and limited social and economic support following divorce. Similarly, previous studies have shown that women may report higher levels of psychological distress and social isolation following marital dissolution, particularly in contexts where divorce is associated with greater social stigma (Sbarra et al., 2015; Badri et al., 2025). Taken together, these findings suggest that cultural norms and gender-related expectations may moderate the relationship between divorce-related experiences and psychological well-being, highlighting the importance of considering sociocultural context when interpreting post-divorce adaptation processes.

Consistent with expectations and prior theoretical frameworks on coparenting (Feinberg, 2003; McHale & Lindahl, 2011; Lamela & Figueiredo, 2016), correlational analyses indicated that positive dimensions of coparenting, including agreement, closeness, support, and endorsement of the partner’s parenting role, were strongly interrelated and associated with higher life satisfaction, whereas negative dimensions, such as conflict exposure and undermining, showed the opposite pattern. These results support the view that coparenting functions as a multidimensional relational system that extends beyond parenting behaviors to influence broader psychological adaptation and subjective well-being (Teubert & Pinquart, 2010; Zhao et al., 2022; Akarçay Ulutaş & Taşkıran, 2024).

In the regression model, only coparenting closeness and psychological distress emerged as significant unique predictors of life satisfaction. The strong positive association between coparenting closeness and life satisfaction suggests that the emotional and relational quality of the post-divorce or post-separation parenting partnership is particularly salient for parents’ global life satisfaction. This finding is consistent with coparenting theory, which emphasizes the emotional alliance between parents as a key relational process associated with parental adaptation following separation (Feinberg, 2003; McHale et al., 2004; McHale & Lindahl, 2011). Similar findings have been reported in previous studies showing that supportive and emotionally connected coparenting relationships are associated with better parental adjustment, lower psychological distress, and higher levels of well-being following divorce (Lamela et al., 2016; Augustijn, 2023; Ferraro & Lucier-Greer, 2022).

An important finding is that only coparenting closeness remained a significant predictor of life satisfaction after controlling for the other coparenting dimensions. Given the moderate to strong intercorrelations among the positive coparenting dimensions, this finding should be interpreted as reflecting the unique variance explained by coparenting closeness after statistically accounting for its overlap with agreement, support, and endorsement, rather than as evidence that these other dimensions are unimportant. This pattern suggests that the emotional quality of the coparenting relationship may be more strongly related to parental well-being than specific behavioral aspects of coparenting. While agreement, support, and endorsement primarily reflect functional coordination in parenting tasks, closeness may capture a broader sense of emotional connection, trust, mutual respect, and relational continuity between former partners. Nevertheless, this finding should be interpreted with caution. The positive coparenting dimensions were moderately to strongly intercorrelated, indicating substantial shared variance among conceptually related constructs. Consequently, the loss of statistical significance for agreement, support, and endorsement in the regression model should not be interpreted as evidence that these dimensions are unimportant. Rather, after accounting for their shared variance, coparenting closeness retained the strongest unique association with life satisfaction. Similar interpretations have been proposed in previous research emphasizing the central role of emotional alliance within the coparenting subsystem and its contribution to parental adjustment and family functioning (Feinberg, 2003; McHale & Lindahl, 2011; Lamela et al., 2016). Furthermore, recent evidence suggests that cooperative and emotionally supportive coparenting relationships are associated with higher parental well-being and life satisfaction following separation, reinforcing the importance of relational quality as a potential resilience-related resource associated with better parental well-being following separation (Augustijn, 2023; Ferraro & Lucier-Greer, 2022). Although these theoretical explanations are consistent with Family Systems Theory and the Stress Process Model, they should be regarded as plausible interpretations rather than conclusions that can be established from the present cross-sectional data. Future longitudinal research is needed to examine whether these proposed processes account for the observed associations over time.

One possible interpretation is that coparenting closeness captures broader relational qualities, including emotional connection, mutual trust, and cooperative engagement between former partners. From a Family Systems perspective, these characteristics have been conceptualized as important components of the coparenting relationship and have consistently been associated with better parental adjustment following separation (Minuchin, 1985; Feinberg, 2003; McHale & Lindahl, 2011; Lamela et al., 2016). However, given the cross-sectional design, this interpretation should be regarded as theoretically informed rather than causal. Furthermore, recent evidence suggests that supportive and emotionally connected coparenting relationships may serve as resources associated with better psychological adjustment that buffer the negative psychological consequences of divorce and promote adaptive post-separation functioning (Ferraro & Lucier-Greer, 2022).

Psychological distress emerged as a significant negative predictor of life satisfaction, highlighting the central role of emotional functioning in post-divorce adaptation. This finding is consistent with a substantial body of literature demonstrating that depressive symptoms, anxiety, and stress are among the strongest correlates of reduced subjective well-being and life satisfaction across diverse populations (Diener et al., 2018; Keyes, 2005; Steel et al., 2008). Within the context of divorce and separation, elevated levels of psychological distress may compromise individuals’ ability to effectively cope with relational, parenting, and daily-life challenges, thereby negatively influencing broader evaluations of life quality and personal adjustment.

The present findings also provide support for the Stress Process Model (Pearlin et al., 1981; Pearlin, 1999), which proposes that chronic interpersonal stressors contribute to psychological maladjustment through the cumulative burden of repeated emotional demands over time. Following separation or divorce, ongoing coparenting difficulties, conflictual interactions, and parenting-related challenges may function as persistent relational stressors that erode psychological resources and increase vulnerability to emotional distress. Consistent with this interpretation, previous research has shown that higher-quality coparenting relationships are associated with better psychological adjustment and more adaptive family functioning following divorce (Bergström et al., 2021; Lamela et al., 2016).

Importantly, the strong association observed between psychological distress and life satisfaction is consistent with the possibility that psychological distress may help explain the relationship between relational experiences and subjective well-being. However, because mediation was not formally tested and the cross-sectional design does not permit conclusions regarding temporal ordering, this interpretation should be considered theoretically informed rather than empirically demonstrated. Previous research has similarly shown that parenting-related stress is associated with lower life satisfaction alongside relational and psychological difficulties (Han et al., 2024), supporting the plausibility of this interpretation. Future longitudinal studies using formal mediation analyses are needed to determine whether psychological distress accounts for the association between coparen ting quality and life satisfaction.

Interestingly, several coparenting dimensions that were significantly correlated with life satisfaction did not retain significance in the multivariate model. This pattern may reflect substantial shared variance among coparenting constructs, particularly given the strong intercorrelations observed between support, endorsement, agreement, and closeness. Previous research has similarly shown that coparenting dimensions often overlap conceptually and empirically, reflecting interconnected aspects of a broader relational system rather than entirely independent constructs (Feinberg, 2003; McHale & Lindahl, 2011; Teubert & Pinquart, 2010). Consequently, when multiple coparenting dimensions are entered simultaneously into regression models, their unique predictive contributions may be attenuated due to shared variance. An alternative explanation is that coparenting closeness retained statistical significance because it captures variance shared with conceptually related positive coparenting dimensions while also reflecting broader relational qualities not fully represented by the remaining subscales. This interpretation is consistent with theoretical models emphasizing the central role of emotional alliance in shaping both family functioning and parental adjustment (Feinberg, 2003; Lamela et al., 2016).

Exposure to conflict and coparenting undermining also lost significance in the regression model despite their significant bivariate associations with life satisfaction and psychological distress. One possible interpretation is that the observed associations between conflict exposure, coparenting undermining, and life satisfaction may partly reflect their shared relationships with psychological distress rather than direct independent associations. However, because mediation was not formally tested and the cross-sectional design precludes conclusions regarding temporal ordering, this interpretation should be regarded as theoretically informed rather than empirically demonstrated. According to the Stress Process Model, chronic interpersonal stressors contribute to reduced well-being by increasing emotional burden and diminishing psychological coping resources over time (Pearlin et al., 1981; Pearlin, 1999). Within post-divorce family systems, conflictual coparenting interactions and undermining behaviors may therefore exert their influence indirectly by increasing distress, which subsequently affects global evaluations of life satisfaction. This interpretation is consistent with previous research demonstrating that interparental conflict is associated with poorer psychological adjustment, elevated stress, and lower well-being following divorce (Lamela et al., 2016; van Dijk et al., 2020; Zhao et al., 2022; M. Wang et al., 2026). Similar observations have been reported in interventions targeting high-conflict coparenting relationships, where reductions in interparental hostility were accompanied by improvements in parental adjustment and relational functioning (Stolnicu et al., 2022). Collectively, these findings suggest that negative coparenting experiences may be more strongly linked to life satisfaction through their impact on emotional functioning than through a direct effect on subjective well-being.

From a theoretical perspective, the findings support integrative models of post-divorce adaptation that emphasize the dynamic interplay between interparental relationships and individual mental health. In particular, the results are consistent with coparenting frameworks proposing that the quality of the coparenting alliance represents a central mechanism through which family processes influence parental adjustment and well-being (Feinberg, 2003; McHale & Lindahl, 2011; Lamela et al., 2016; H. Wang, 2022; M. Wang et al., 2026).

The finding that coparenting closeness emerged as a unique predictor of life satisfaction further reinforces the importance of the emotional dimension of the coparenting relationship in shaping post-divorce adaptation. Moreover, the strong predictive role of psychological distress highlights the need to conceptualize coparenting within broader models of stress, coping, and mental health rather than as an isolated relational construct (Pearlin et al., 1981; Pearlin, 1999). Taken together, the findings suggest that relational and psychological processes operate in parallel and interactively in predicting life satisfaction following separation and divorce. This interpretation is also consistent with recent longitudinal evidence indicating substantial heterogeneity in post-divorce adaptation trajectories, suggesting that individual and contextual resources may shape long-term adjustment following marital dissolution (Leopold & Kalmijn, 2024).

Although respondents may have varying subjective interpretations of what constitutes closeness, this study relied on a standardized instrument to establish a uniform operational baseline. By assessing specific, observable behaviors and perceived support, rather than relying on abstract self-definitions, the measurement instrument minimized individual variance in how “closeness” was conceptualized. However, it is important to recognize that the psychological significance of closeness in coparenting may still be influenced by individual expectations, the relationship between former life partners, socio-financial aspects, and cultural or gendered customs regarding parenting roles. For example, in Romanian society, divorced or single parents are not stigmatized or victimized. Women could be financially independent from their former partners, integrated into society, and professionally successful.

The unique significance of coparenting closeness should not be considered as a evidence that is the more vital to family functioning than agreement, support or endorsement. Our findings showed that closeness provided a distinct contribution to the model even after accounting for the robust, overlapping effect of the other subscales. Agreement, support and endorsement remain foundational, and the significance of closeness highlights its distinctive value rather than dominance.

The present results are also broadly consistent with findings reported across different cultural contexts. Studies conducted in Portugal (Lamela et al., 2016), Spain (Pellón et al., 2024), and Türkiye (Çelik & Nazlı, 2025) similarly highlight the importance of post-divorce relational functioning for parental adjustment and well-being. Although cultural norms surrounding divorce, parenting, and family roles vary substantially across societies, positive coparenting processes appear to be consistently associated with better psychological adaptation across cultural contexts. Furthermore, recent evidence from both Western and non-Western contexts suggests that supportive coparenting relationships have been associated with greater resilience, reduce emotional distress, and facilitate adaptive adjustment following family transitions (Augustijn, 2023; Badri et al., 2025). Taken together, these findings are consistent with the possibility that maintaining constructive and emotionally supportive coparenting relationships may be associated with better psychological adaptation following separation across diverse cultural settings. Nevertheless, because cultural variables were not directly assessed in the present study, these interpretations should be regarded as tentative and warrant further investigation in cross-cultural research.

*Cultural and practical implications*: Our results have several practical implications for clinical and family interventions targeting divorced or separated parents. First, the present findings suggest that interventions may benefit from strengthening coparenting closeness alongside efforts to reduce interparental conflict. Recent evidence further suggests that both face-to-face and online psychoeducational programs may contribute to improving coparenting functioning and parental adjustment following divorce, highlighting the potential value of accessible intervention formats for separated families (Ordóñez-Carabaño et al., 2026). Programs such as coparenting counseling or structured mediation may consider incorporating components aimed at fostering emotional cooperation and mutual recognition between former partners. The present findings identify these domains as potentially relevant targets for future program development and evaluation rather than as established intervention effects. Second, the strong negative association between psychological distress and life satisfaction suggests that mental health support should be integrated into coparenting interventions. Addressing symptoms of anxiety, depression, and general distress may represent a promising component of future intervention programs designed to support post-divorce adjustment. However, longitudinal and intervention studies are needed to determine whether reducing psychological distress contributes to subsequent improvements in life satisfaction. The lack of unique effects for several coparenting dimensions suggests that future intervention programmes may benefit from adopting a broader systemic perspective that addresses overall coparenting quality rather than focusing exclusively on isolated behavioral dimensions (e.g., agreement or support). Whether such an approach improves parental outcomes should be established in future longitudinal and intervention research. Overall, the present findings should be interpreted as identifying theoretically and clinically relevant domains for future programme development rather than demonstrating the effectiveness of specific intervention strategies.

*Limitations of the study*: Several limitations should be acknowledged. The cross-sectional design precludes causal inferences regarding the relationships among coparenting, psychological distress, and life satisfaction. Although the present findings are consistent with theoretical expectations and prior empirical work, they do not allow conclusions regarding directionality. Future longitudinal research would be needed to examine temporal ordering and potential reciprocal associations between coparenting quality and psychological adaptation in terms of life satisfaction following separation. Additionally, the reliance on self-report measures may introduce shared method variance and social desirability bias. This is particularly relevant in the context of coparenting, where participants may underreport negative interactions (e.g., conflict, undermining) or overreport adaptive dimensions (e.g., support, agreement). Future studies could benefit from incorporating observational methods or diary-based assessments to capture more ecologically valid indicators of coparenting dynamics. Another important limitation concerns the sampling strategy. Participants were recruited using convenience sampling through online social media platforms rather than probability-based sampling methods. Consequently, the sample may not be representative of the broader population of divorced or separated parents in Romania, particularly individuals with lower digital literacy, limited internet access, or reduced engagement in online parenting communities. Therefore, the generalizability of the findings should be interpreted with caution. Future studies would benefit from using more diverse recruitment strategies and probability-based sampling procedures whenever feasible. In addition, the sample was predominantly composed of highly educated participants living in urban areas, which may further limit the generalizability of the findings to more socioeconomically and geographically diverse populations. Furthermore, it should also be noted that the causes of separation or divorce were self-reported and categorized into broad groups (e.g., marital conflict, infidelity, incompatibility). These categories may oversimplify complex relational processes underlying union dissolution and may be subject to retrospective bias. Participants differed with respect to their separation status, including individuals who were divorced, formally separated, or undergoing separation proceedings. These groups may differ in their psychological adjustment and coparenting experiences, and future studies should examine these stages separately. Another limitation is that information was obtained from only one parent in each family. Because coparenting is inherently relational, relying on a single informant may not fully capture the mutual and interactive nature of the coparenting relationship. Future studies would benefit from dyadic designs including reports from both former partners to provide a more comprehensive assessment of coparenting dynamics. In addition, the marked predominance of women in the sample limits the extent to which the findings can be generalized to fathers. Mothers and fathers may differ in their coparenting experiences, psychological adjustment, and perceptions of post-divorce family relationships. Consequently, the observed associations may primarily reflect maternal experiences following separation or divorce. Future studies should therefore recruit more gender-balanced samples and examine gender not only as a control variable but also as a potential moderator of the associations among coparenting quality, psychological distress, and life satisfaction. The present study also did not collect detailed information regarding child custody arrangements, parenting time, frequency of parent–child contact, or legal custody agreements. The family characteristics may influence both coparenting quality and parental well-being and should therefore be incorporated into future research. In addition, future studies should consider incorporating socioeconomic indicators, such as household income or financial strain, together with more detailed information regarding post-divorce contact patterns between former partners, as these variables may further contribute to explaining variability in parental adjustment and life satisfaction. Finally, the present study was conducted within a Romanian cultural context, which may be characterized by specific norms regarding parenting roles, post-divorce family arrangements, and perceptions of parental conflict. Because these contextual factors were not directly assessed, their potential influence should be interpreted cautiously.

Cross-cultural studies are therefore needed to examine the extent to which these findings generalize to other sociocultural contexts. Comparative research could also clarify how cultural norms regarding gender roles and family structure influence coparenting quality and its association with psychological life satisfaction.

## 5. Conclusions

The present study underscores the importance of both relational and psychological factors in understanding life satisfaction among post-divorce and post-separation parents. Coparenting closeness and psychological distress emerged as central predictors among Romanian divorced and separated parents, highlighting that life satisfaction in this context is shaped by the emotional quality of the coparenting relationship and individual mental health.

## Figures and Tables

**Table 1:** Sample characteristics *.

	*N*	%
Gender		
Women	172	83.5%
Men	34	16.5%
Education level		
Gymnasium	3	1.5%
High school	20	9.7%
Vocational school	18	8.7%
Bachelor’s degree	84	40.8%
Master’s degree	70	34%
PhD	9	4.4%
Post-doctoral training	2	1%
Residential area		
Urban	175	85%
Rural	31	15%
Marital status		
Divorced	151	73.3%
Separated	26	12.6%
In the process of divorce	29	14.1%
Child’s living arrangement		
Lives with me	171	83%
Lives with the other parent	35	17%
Relationship status		
Single	121	58.7%
In a committed romantic relationship	85	41.3%
Causes of divorce/separation		
Infidelity	41	19.9%
Marital conflict	59	28.6%
Incompatibility	36	17.5%
Lack of involvement	12	5.8%
Alcohol abuse	15	7.3%
Physical violence	20	9.7%
Abuse	11	5.3%
Emotional distancing	12	5.8%

* Number and percentage (*N* and %).

**Table 2:** Descriptive statistics for instruments.

	Min	Max	M	SD	Skewness (SE)	Kurtosis (SE)
Coparenting Agreement	0	6	2.56	1.74	0.04 (0.17)	−1.06 (0.34)
Coparenting Closeness	0	6	2.48	1.53	0.47 (0.17)	−0.74 (0.34)
Exposure to conflict	0	6	2.40	1.81	0.52 (0.17)	−0.74 (0.34)
Coparenting support	0	6	2.42	1.91	0.37 (0.17)	−1.12 (0.34)
Coparenting undermining	0	6	2.80	1.73	0.17 (0.17)	−1.06 (0.34)
Endorse partner parenting	0	6	2.87	1.80	0.08 (0.17)	−1.22 (0.34)
Depression	0	21	5.34	5.28	1.06 (0.17)	0.41 (0.34)
Anxiety	0	21	5.97	5.63	0.95 (0.17)	0.24 (0.34)
Stress	0	21	7.01	5.99	0.76 (0.17)	−0.39 (0.34)
Psychological distress (total)	0	63	18.32	16.00	0.03 (0.17)	0.18 (0.34)
Satisfaction with life	5	35	23.87	5.91	−0.61 (0.17)	−0.002 (0.34)

**Table 3:** Pearson correlations between study variables.

	1	2	3	4	5	6	7	8	9	10	11	12	13
1. Coparenting Agreement	-												
2. Coparenting Closeness	0.58 **	-											
3. Exposure to conflict	−0.33 **	−0.28 **	-										
4. Coparenting support	0.66 **	0.84 **	−0.34 **	-									
5. Coparenting undermining	−0.73 **	−0.41 **	0.43 **	−0.53 **	-								
6. Endorse partner parenting	0.68 **	0.73 **	−0.29 **	0.79 **	−0.46 **	-							
7. Psychological distress	−0.06	−0.12	0.45 **	−0.09	0.20 **	−0.02	-						
8. Depression	−0.02	−0.15 *	0.40 **	−0.08	0.18 *	−0.05	0.95 **	-					
9. Anxiety	−0.09	−0.07	0.47 **	−0.10	0.22 **	−0.06	0.95 **	0.86 **	-				
10. Stress	−0.07	−0.12	0.41 **	−0.07	0.16 *	0.04	0.94 **	0.83 **	0.84 **	-			
11. Satisfaction with life	0.22 **	0.44 **	−0.34 **	0.34 **	−0.23 **	0.33 **	−0.48 **	−0.49 **	−0.40 **	−0.48 **	-		
12. Age	0.05	−0.007	0.05	0.03	−0.12	−0.05	0.04	0.05	0.006	0.05	0.03	-	
13. Time since separation/divorce	−0.03	−0.03	−0.07	−0.05	−0.11	−0.11	−0.25 **	−20 **	−0.26 **	−0.24 **	0.04	0.29 **	-

* *p* < 0.05, ** *p* < 0.01.

**Table 4:** Multiple regression analysis.

Predictors	B	SE	B	T	*p*	Lower 95% CI	Upper 95% CI	Tolerance	VIF
Intercept (constant)	20.870	2.815		7.413	<0.001	15.317	26.422		
Coparenting Agreement	−0.372	0.337	−0.110	−1.103	0.271	−1.036	0.293	0.309	3.237
Coparenting Closeness	1.676	0.405	0.434	4.137	<0.001	0.877	2.474	0.278	3.602
Exposure to conflict	−0.212	0.225	−0.065	−0.943	0.347	−0.655	0.231	0.645	1.549
Coparenting support	−0.486	0.378	−0.157	−1.287	0.200	−1.231	0.259	0.205	4.877
Coparenting undermining	−0.076	0.306	−0.022	−0.248	0.804	−0.680	0.528	0.380	2.629
Endorse partner parenting	0.559	0.363	0.170	1.539	0.126	−0.157	1.275	0.250	3.997
Psychological distress	−0.160	0.024	−0.434	−6.650	<0.001	−0.208	−0.113	0.718	1.392
Gender	−0.138	1.034	−0.009	−0.134	0.894	−2.177	1.901	0.720	1.388
Age	0.084	0.057	0.090	1.477	0.141	−0.028	0.196	0.822	1.217
Time since separation/divorce	−0.010	0.007	−0.080	−1.325	0.187	−0.025	0.005	0.830	1.205

Note. *N* = 206. Gender was dummy coded (0 = women, 1 = men).

## Data Availability

Data Availability Statements upon request to the main author.
